# Efficacy of Hi-Lo Evac Endotracheal Tube in Prevention of Ventilator-Associated Pneumonia in Mechanically Ventilated Poisoned Patients

**DOI:** 10.1155/2016/4901026

**Published:** 2016-08-29

**Authors:** Ahmad Ghoochani Khorasani, Shahin Shadnia, Mohammad Mashayekhian, Mitra Rahimi, Abbas Aghabiklooei

**Affiliations:** ^1^Department of Clinical Toxicology, Loghman-Hakim Hospital, School of Medicine, Shahid Beheshti University of Medical Sciences, Tehran, Iran; ^2^Department of Medical Sciences, Amin Police University, Tehran, Iran; ^3^Department of Legal Medicine, Iran University of Medical Sciences, Tehran, Iran

## Abstract

*Background*. Ventilator-associated pneumonia (VAP) is the most common health care-associated infection. To prevent this complication, aspiration of subglottic secretions using Hi-Lo Evac endotracheal tube (Evac ETT) is a recommended intervention. However, there are some reports on Evac ETT dysfunction. We aimed to compare the incidence of VAP (per ventilated patients) in severely ill poisoned patients who were intubated using Evac ETT versus conventional endotracheal tubes (C-ETT) in our toxicology ICU.* Materials and Methods*. In this clinical randomized trial, 91 eligible patients with an expected duration of mechanical ventilation of more than 48 hours were recruited and randomly assigned into two groups: (1) subglottic secretion drainage (SSD) group who were intubated by Evac ETT (*n* = 43) and (2) control group who were intubated by C-ETT (*n* = 48).* Results*. Of the 91 eligible patients, 56 (61.5%) were male. VAP was detected in 24 of 43 (55.8%) patients in the case group and 23 of 48 (47.9%) patients in the control group (*P* = 0.45). The most frequently isolated microorganisms were* S. aureus* (54.10%) and* Acinetobacter* spp. (19.68%). The incidence of VAP and ICU length of stay were not significantly different between the two groups, but duration of intubation was statistically different and was longer in the SSD group. Mortality rate was less in SSD group but without a significant difference (*P* = 0.68).* Conclusion*. The SSD procedure was performed intermittently with one-hour intervals using 10 mL syringe. Subglottic secretion drainage does not significantly reduce the incidence of VAP in patients receiving MV. This strategy appears to be ineffective in preventing VAP among ICU patients.

## 1. Introduction

Ventilator-associated pneumonia (VAP) is the most common health care-associated infection defined as the involvement of the lung parenchyma that occurs more than 48 hours after intubation and initiation of mechanical ventilation (MV) [1, 2]. VAP increases intensive care unit (ICU) and hospital length of stay and increases costs by at least six days and £12 000 per patient episode [2]. High morbidity and mortality rates of VAP patients have already been reported in several investigations [1–6]. Due to the association of VAP and poor outcomes, VAP prevention is an important topic on the research agenda of ICU clinicians in the past three decades [5]. Microscopic aspiration of subglottic contaminated secretions into the lower respiratory tract is considered as an important mechanism in the pathogenesis of VAP [3]. Impairment of laryngeal function by the endotracheal tube is the particular risk among intubated patients receiving MV [4]. Therefore, VAP prevention should be focused on aspects of care affecting this process.* Staphylococcus aureus* (*S. aureus*) is the main pathogen responsible for VAP and the leading cause of death among all ICU-acquired infections [6]. A preventive method to avoid the progression of subglottic secretions into the lower respiratory tract is subglottic secretion drainage (SSD) which removes secretions using Hi-Lo Evac endotracheal tube (Hi-Lo Evac; Mallinckrodt, Athlone, Ireland; Evac ETT) [7]. Evac ETT is a new and special tracheal tube with a separate dorsal suction lumen which is used for evacuation of subglottic secretions. The suction lumen of Evac ETT has two ports: a subglottic port located 15 mm above the cuff with an elliptical shape (major axis: 6 mm, minor axis: 3 mm) and an external port for connection to suction [8]. Although Evac ETT has been suggested to reduce the risk of development of VAP [9–13], its failure has been reported in some surveys [8, 14] as Girou and colleagues reported that SSD had not decreased the airway colonization [15]. The aim of the present study was to compare the incidence of VAP in severely ill poisoned patients who had been intubated by Evac ETT versus conventional endotracheal tubes (C-ETT) in our toxicology ICU (TICU).

## 2. Materials and Methods

### 2.1. Study Design

This randomized clinical trial was conducted on poisoned patients admitted to our 16-bed TICU of Loghman Hakim Hospital Poison Center (LHHPC) from August 2012 to March 2013. The study was approved by the research ethics committee in Research Deputy Department of the Shahid Beheshti University of Medical Sciences, Tehran, Iran.

### 2.2. Intervention

Patients older than 18 years of age poisoned by any medication or chemical agent who needed intubation and MV and had been admitted to our TICU within the period of the study were included. Indications for intubation were profound disturbance in consciousness with the inability to protect the airway, severe pulmonary or multisystem injury associated with respiratory failure, loss of gag/cough reflex, airway obstruction, and anticipated loss of control of the airway. Indications for MV were ventilation and oxygenation failure. Those younger than 18 years, patients with the history of acquired immunodeficiency syndrome, lung cancer, chronic obstructive pulmonary disease, immunosuppressive therapy, and pregnancy, and cases who had received antibiotics within the last 24 hours before admission were excluded.

Finally, 100 eligible patients with an expected duration of mechanical ventilation of more than 48 hours were recruited in the study and randomly assigned into two groups: (1) subglottic secretion drainage (SSD) group who were intubated by Evac ETT (case group; *n* = 50) and (2) control group who were intubated by conventional ETT (*n* = 50). The SSD procedure was performed intermittently with one-hour intervals using a 10 mL syringe. In both groups, internal diameter of the ETT was 7 to 7.5 mm for women and 8 to 8.5 mm for men and the ETT was inserted to the 23 cm line in men and 21 cm line in women. Neither oral chlorhexidine nor oral antibiotic pastes were administered. All patients received pantoprazole for stress ulcer prophylaxis. All cases were followed up every day by a well-trained ICU nurse until discharge from the TICU.

### 2.3. Vigilance against Microbiological Threats: Diagnosis of VAP

Diagnosis of VAP was confirmed by radiographic lung infiltration with at least two of the following criteria: (1) core temperature higher than 38°C or lower than 35°C, (2) leukocyte count higher than 10000/*μ*L or lower than 4000/*μ*L, (3) presence of new purulent respiratory secretion or any changes in the sputum, (4) significant quantitative culture of respiratory secretions by tracheal aspirate (>106 cfu/mL), (5) detection of rales or dullness on chest examination, and (6) at least 10% decrease in arterial pO2 [7, 16]. Diagnosis and treatment of VAP was made by an infectious disease specialist who was blind to the treatment assignment.

### 2.4. Variables Recorded

Demographic characteristic and clinical and paraclinical data including age, gender, mental status by Glasgow coma scale (GCS), type of poisoning, time elapsed between exposure and admission, on-admission vital signs, tracheotomy, reintubation, and type and duration of antibiotic therapy were recorded in a self-made questionnaire.

Incidence of VAP (per ventilated patients), duration of mechanical ventilation, length of ICU stay, length of hospital stay, and mortality rate were also recorded as the outcome measurements of the patients and evaluated.

### 2.5. Statistical Analysis

Normality of data in quantitative variables was checked by Shapiro-Wilk test and data Q-Q plot. To evaluate the changes between groups in nominal variables, we used Chi-square or Fisher's exact test. *t*-test and Mann-Whitney *U* test were used to evaluate the difference between groups in quantitative variables. A *P* value less than 0.05 was considered to be statistically significant. Statistical analysis was performed by statistical package for social sciences (SPSS) version 21.0 (IBM Co, Chicago, Ill, USA).

## 3. Results

Seven and two patients in the case and the control group self-extubated themselves in less than 24 hours, did not need reintubation, and were therefore excluded. A total of 91 patients (43 in the Evac-ETT and 48 in the C-ETT groups) were finally included, of whom 56 (61.5%) were male and 35 (38.5%) were female. Mean age was 28.64 ± 12.05 and 38.66 ± 14.98 years in case and control groups, respectively, Table 1. There was no significant difference between these two groups regarding age, sex, time elapsed between exposure and admission, vital signs on admission, and type of poisoning (all *P* values were higher than 0.05), Table 2. Median GCS was 7 and 8 in the case and control groups, respectively. Mean duration of intubation was 4.5 ± 3.9 days (median [IQR]: 3 days [0.3–14 days]) and 3 ± 2.5 days (median [IQR]: 2 days [0.3–11 days]) in case and control groups, respectively (*P* = 0.03), Table 1. Accidental extubation was reported in 11 cases and 21 controls (*P* = 0.037). Three patients in each group were reintubated during the study period. Mean ICU length of stay was 7 ± 6.3 days in cases (median [IQR]: 5 days [0.5–30 days]) and 4.8 ± 4.7 days in controls (median [IQR]: 3.1 days [0.7 to 30 days]; *P* = 0.98). VAP was detected in 24 of 43 (55.8%) patients in the case group and 23 of 48 (47.9%) patients in the control group (*P* = 0.45), Table 1. VAP incidence and ICU length of stay were not significantly different between the two groups, but duration of intubation was. In 47 of the available specimens, a total of 60 bacterial isolates grew. Quantitative cultures reached the 106 cfu/mL (threshold of positivity for respiratory secretions) in 47 (51.65%) patients (positive cases) and were below this threshold in 44 (48.35%) patients (negative cases). The cultures were mono-microbial in 35 patients yielding 35 isolates and poly-microbial in 13 patients yielding 26 isolates. Mean duration of antibiotic therapy was 6.3 ± 2.4 and 5.7 ± 2.1 days in case and control groups, respectively. Tracheostomy was performed in three patients and one of them was in the case group. Mortality rate was 4.7% and 8.3% in the cases and controls. Although mortality rate was less in the case group, this difference was statistically insignificant (*P* = 0.68).

## 4. Discussion

VAP is the leading cause of poor outcome, morbidity, and high mortality rate in ICUs [1]. The European Prevalence of Infection in the Intensive Care study reported that VAP was 45% of all ICU-acquired infections in European ICUs [17]. Differences in the study population, definitions, and type of hospital or ICU were reasons of the wide VAP incidence variation from 7% to 70% [1]. In the present study, most of the patients were male and young which is completely predictable in TICU [1].

We used quantitative cultures of the homogenized EA. Despite newer invasive bronchoscopic methods available to diagnose VAP, many physicians use quantitative culture of the endotracheal aspirates with a cut-off point of 106 cfu/mL. Good correlations have been found between the results of quantitative cultures of protected specimen brush or bronchoalveolar lavage and those of quantitative cultures of EA [18]. According to our results, the most frequently isolated microorganisms were* S. aureus* (54.10%) and* Acinetobacter* spp. (19.68%). This is in accordance with previous studies which showed that* S. aureus* was the most common isolate from ICU patients with nosocomial pneumonia [19–21].

The incidence of VAP was 55.8% (per ventilated patients) in cases and 47.9% in controls (*P* > 0.05). Comparison of the other outcomes such as ICU length of stay and mortality rate showed no significant difference between these two groups, either. Likewise, three other studies found that SSD did not decrease the incidence of VAP or airway colonization [15, 22–24]. In a randomized controlled trial on 343 cardiac surgery patients, no significant difference was found in frequency of VAP with or without continuous microaspiration of subglottic secretions (CASS).

There were no differences in hospital mortality, overall duration of MV, or length of hospital stay, either, although episode of VAP occurred significantly later in the group receiving CASS compared to the group without CASS [22]. Subglottic drainage, however, was shown to reduce VAP rates in some other studies; therefore, these investigations suggested using an orotracheal tube that enabled continuous or intermittent subglottic suctioning, especially in patients who might require intubation for 48 hours or longer [23, 24]. A meta-analysis in 2005 evaluated 896 patients from five surveys and showed 49-percent reduction in risk of VAP using SSD but without any differences in other outcomes. This study also suggested that SSD might not be effective in patients requiring less than 72 hours of MV likely because several days are required for the accumulation of contaminated subglottic secretions and their subsequent microaspiration [9].

However, major disadvantages such as failure to aspirate subglottic secretions with the Evac ETT and problems with airway mucosal tissue were mentioned in some other investigations [8, 14, 15]. Lack of a fiber-optic bronchoscope for evaluating the subglottic part of the suction lumen was a limitation of the current study. Another limitation was that we did not compare the airway mucosal injury between the two groups. Blinding process was another limitation because Evac and conventional ETT are visually different and the physicians could not be blinded to the groups; however, our infectious disease specialist was blinded to the procedure of the study.

## 5. Conclusion

The SSD procedure was performed intermittently with one-hour intervals using 10 mL syringe. Subglottic secretion drainage does not significantly reduce the incidence of VAP in patients receiving MV. This strategy appears to be ineffective in preventing VAP among T ICU patients.

## Figures and Tables

**Table 1:** Results.

Variables	Case group (Evac ETT)	Control group (C-ETT)	*P* value
Mean duration of intubation (days)	4.5 ± 3.9	3 ± 2.5	0.03
Accidental extubation	11	21	0.037
Mean ICU length of stay	7 ± 6.3 days	4.8 ± 4.7	0.98
VAP	55.8%	47.9%	0.45
Mean age (years)	28.64 ± 12.05	38.66 ± 14.98	

**Table 2:** Comparison of sex, age, vital signs on admission, and time elapsed between exposure and admission between the case and control groups.

Variables	Case group (Evac ETT)	Control group (C-ETT)	*P* value
Male	26 (60.5%)	30 (62.5%)	0.84^*∗*^
Female	17 (39.5%)	(37.5%)	
Age	35.4 ± 13.7	32.2 ± 15.2	0.16^‡^
Systolic blood pressure	109 ± 20	117 ± 25	0.09^†^
Temperature	37 ± 1	37 ± 1	0.97^‡^
Pulse rate	93 ± 21	99 ± 26	0.2^†^
Time elapsed between exposure and admission	5.7 ± 6.2	4.5 ± 4.7	0.27^‡^

^†^Based on *t*-test. ^‡^Based on Mann-Whitney test. ^*∗*^Based on Chi-square test.
